# Detecting Influenza A(H5N1) Viruses through Severe Acute Respiratory Infection Surveillance, Cambodia

**DOI:** 10.3201/eid3203.250832

**Published:** 2026-03

**Authors:** William W. Davis, Kathrine R. Tan, Borann Sar, Alyssa Finlay, Heng Seng, Vicheth Long, Savuth Chin, Darapheak Chau, Kim Sreng Leang, Mich Vann, Sovandara Lam, Dara Chan, Ly Vannara Tek, Sovann Ly, Timothy M. Uyeki

**Affiliations:** US Centers for Disease Control and Prevention, Atlanta, Georgia, USA (W.W. Davis, K.R. Tan, B. Sar, A. Finlay, T.M. Uyeki); US Centers for Disease Control and Prevention, Phnom Penh, Cambodia (H. Seng, V. Long, S. Ly); Cambodia National Institute of Public Health, Phnom Penh (S. Chin, D. Chau); National Pediatric Hospital, Phnom Penh (K.S. Leang); Khmer Soviet Hospital, Phnom Penh (M. Vann); Kampot Provincial Hospital, Kampot, Cambodia (S. Lam); Svay Rieng Provincial Hospital, Svay Rieng, Cambodia (D. Chan); Kuntha Bopha Phnom Penh Hospital, Phnom Penh (L.V. Tek)

**Keywords:** influenza, viruses, respiratory infections, zoonoses, severe acute respiratory infection, SARI, influenza A virus, H5N1, public health surveillance, Cambodia

## Abstract

Of 19 human cases of avian influenza A(H5N1) virus infection detected during January 2023–March 2025 in Cambodia, 12 (63%) were detected directly by surveillance for severe acute respiratory infection (SARI) or indirectly by testing ill close contacts. SARI surveillance can supplement other surveillance sources for identifying H5N1 cases.

Novel influenza A viruses have pandemic potential, and early identification of infections in humans is crucial for rapid response and containment. Detection of human infection with a novel influenza A virus initiates response activities, including antiviral postexposure prophylaxis for close contacts, symptom monitoring, and implementation of interventions to reduce transmission risk. In addition, timely clinical suspicion and early diagnosis of novel influenza A virus infections in humans are critical for optimizing clinical management: the Centers for Disease Control and Prevention recommend persons with suspected novel influenza A virus infection be promptly isolated, tested for influenza A viruses, and started on empiric antiviral treatment without waiting for testing results (*1*).

The primary goal of sentinel surveillance for severe acute respiratory infection (SARI) (*2*) is to detect trends in severe seasonal influenza and other respiratory viruses (*3*). Typically, surveillance sites are referral hospitals where a subset of patients meeting the SARI case definition (acute respiratory illness causing temperature >38°C and cough that has onset within the previous 10 days and requires hospitalization) (*2*) have respiratory specimens collected and tested for seasonal and (if negative) novel influenza A viruses. A reliably functioning SARI surveillance system for seasonal influenza also provides an infrastructure for detecting novel influenza A virus infections, including rapid identification of severely ill patients, shipping and testing of specimens, and reporting chains for response.

During February 2023–March 2025, a total of 19 human cases of influenza A(H5N1) were identified in Cambodia. Nine (47%) of the 19 cases were detected by SARI surveillance; 3 (16%) additional cases that involved exposure to sick or dead poultry were identified by testing close contacts of those cases. Investigations concluded that findings were most consistent with poultry-to-human transmission, although in some cases human-to-human transmission could not be completely ruled out. Of the remaining 7 identified cases, 6 (26%) were suspected by clinicians on the basis of patients’ history of exposure to sick or dead poultry, 1 (5%) was found through active case finding in response to one of those cases, and 1 (5%) was diagnosed by postmortem testing. Of the 19 H5N1 patients, 14 were <18 years of age; 7 pediatric cases were identified at SARI surveillance sites, and 3 of those 7 were detected at the National Pediatric Hospital in Phnom Penh. A highly pathogenic avian influenza A(H5N1) virus isolated from 1 patient was selected as an influenza A(H5) vaccine candidate (*4*).

Compared with the 5 cases diagnosed by clinicians, the 9 cases detected by SARI surveillance were diagnosed earlier (time from symptom onset to diagnosis: median 6 [range 3–9] days vs. median 8 [range 4–12] days) and treated with oseltamivir earlier (time from symptom onset to initiation of treatment: median 5 [range 2–7] days vs. median 7 [range 4–12] days). Oseltamivir treatment of H5N1 patients is associated with survival when initiated within 2 days of illness onset (*5*); however, most cases identified by SARI surveillance were identified much later. All but 1 of the 14 case-patients sought care at other healthcare facilities before seeking care at the hospital where they were diagnosed with influenza A(H5N1), highlighting an urgent need for clinician education to identify cases and start oseltamivir treatment as soon as possible.

SARI surveillance has limitations in detecting novel influenza A virus infections. Some novel influenza A case-patients might not develop SARI, and others with SARI might seek care at a private clinic that may not have influenza testing. Because SARI surveillance tests a subset of eligible SARI patients and does not usually have national coverage, H5N1 virus infections will be missed (*6*,*7*). SARI surveillance systems are costly, detections of novel influenza A viruses are rare (*8*)—in Cambodia, roughly 1 H5N1 virus was detected per 1,100 SARI cases (Table)—and other forms of surveillance are effective for detecting novel influenza A infections. For example, clinician suspicion led to diagnosis of 5 (25%) of 19 H5N1 cases in Cambodia during the study period. Thus, a combination of sentinel and event-based surveillance systems ideally should be used to detect novel influenza A viruses (*9*). Training clinicians at private clinics, outpatient settings, and nonsentinel site hospitals on the manifestations, diagnosis, and reporting of novel influenza A virus infections and expanding access to influenza testing, including for influenza A(H5) viruses, especially in areas with novel influenza A viruses circulating among poultry and other animals, is essential to improving detection of human cases.

**Table:** SARI cases and influenza A and influenza A(H5N1) virus detections in humans, by surveillance site, Cambodia, January 2023–March 2025*

Detections	Surveillance site									Total
	AHC	CCH	KCH	KPH	KSH	KVH	NPH	PKH	SVH	
SARI cases	1,078	547	888	521	1,168	779	2,403	1,635	1,346	10,365
Influenza A virus detections	62	87	67	49	141	103	201	119	94	923
H5N1 virus detections	0	0	0	1	3	1	3	0	1	9

*All patients with SARI were tested for influenza. AHC, Angkor Hospital for Children, Siem Reap, Cambodia; CCH, Chey Chum Nas Hospital, Ta Khmau, Cambodia; KCH, Kampong Cham Hospital, Kampong Cham, Cambodia; KPH, Kampot Hospital, Kampot, Cambodia; KSH, Khmer Soviet Hospital, Phnom Penh, Cambodia; KVH, Kirivong Hospital, Takeo Province; NPH, National Pediatric Hospital, Phnom Penh; PKH, Preah Kosomak Hospital, Phnom Penh; SARI, severe acute respiratory infection; SVH, Svay Rieng Hospital, Svay Rieng, Cambodia.

In conclusion, during February 2023–March 2025, sentinel SARI surveillance in Cambodia directly or indirectly detected 63% (12/19) of human cases of influenza A(H5N1). To improve surveillance coverage and decrease times from symptom onset to detection, countries should consider multilayered surveillance systems, including event-based surveillance and educating clinicians on diagnosis of novel influenza A, especially in areas with high zoonotic transmission risk (*10*).
